# A systematic review of simulation studies which compare existing statistical methods to account for non-compliance in randomised controlled trials

**DOI:** 10.1186/s12874-023-02126-w

**Published:** 2023-12-16

**Authors:** Lucy Abell, Francesca Maher, Angus C Jennings, Laura J Gray

**Affiliations:** Department of Population Health Sciences, University of Leicester, Leicester, UK

**Keywords:** Non-compliance, Simulation studies, Statistical methods, Randomised controlled trials

## Abstract

**Introduction:**

Non-compliance is a common challenge for researchers and may reduce the power of an intention-to-treat analysis. Whilst a per protocol approach attempts to deal with this issue, it can result in biased estimates. Several methods to resolve this issue have been identified in previous reviews, but there is limited evidence supporting their use. This review aimed to identify simulation studies which compare such methods, assess the extent to which certain methods have been investigated and determine their performance under various scenarios.

**Methods:**

A systematic search of several electronic databases including MEDLINE and Scopus was carried out from conception to 30th November 2022. Included papers were published in a peer-reviewed journal, readily available in the English language and focused on comparing relevant methods in a superiority randomised controlled trial under a simulation study. Articles were screened using these criteria and a predetermined extraction form used to identify relevant information. A quality assessment appraised the risk of bias in individual studies. Extracted data was synthesised using tables, figures and a narrative summary. Both screening and data extraction were performed by two independent reviewers with disagreements resolved by consensus.

**Results:**

Of 2325 papers identified, 267 full texts were screened and 17 studies finally included. Twelve methods were identified across papers. Instrumental variable methods were commonly considered, but many authors found them to be biased in some settings. Non-compliance was generally assumed to be all-or-nothing and only occurring in the intervention group, although some methods considered it as time-varying. Simulation studies commonly varied the level and type of non-compliance and factors such as effect size and strength of confounding. The quality of papers was generally good, although some lacked detail and justification. Therefore, their conclusions were deemed to be less reliable.

**Conclusions:**

It is common for papers to consider instrumental variable methods but more studies are needed that consider G-methods and compare a wide range of methods in realistic scenarios. It is difficult to make conclusions about the best method to deal with non-compliance due to a limited body of evidence and the difficulty in combining results from independent simulation studies.

**PROSPERO registration number:**

CRD42022370910.

**Supplementary Information:**

The online version contains supplementary material available at 10.1186/s12874-023-02126-w.

## Background

Non-compliance (also referred to as non-adherence) to the intervention is a type of protocol deviation which occurs when participants in clinical trials do not adhere to the protocol of the intervention group that they were originally randomised to and may refer to individuals in all arms dropping out or missing certain elements of their randomised intervention. Analysis in the presence of non-compliance is a common challenge for researchers, with the average rate across disease areas found to be almost 25% in a review of 569 trials [1]. Addressing this issue and ignoring original randomisation means that it cannot be guaranteed that the relationship between intervention and outcome is unconfounded [2]. However, non-compliance also has the potential to reduce the power of the gold standard intention-to-treat (ITT) analysis ​ [3]​, in which participants are analysed based on their allocated group, irrespective of the intervention they actually received. These limitations are summarised nicely by Sagarin et al. (2014), who remark that “non-compliance is difficult to model and perilous to ignore” [4]​.

Despite this clear issue, there is a distinct lack of guidance surrounding the handling and reporting of non-compliance within randomised controlled trials (RCTs), with the 2010 CONSORT guidelines stating that “the simple way to deal with any protocol deviations is to ignore them” [5]​. These guidelines recommend the reporting of an effect size estimated using per protocol (PP) methods, where non-compliers are excluded from analysis, in addition to reporting of the ITT effect. Whilst approaches such as PP and as-treated (AT) - where participants are classified by the treatment they received rather than the one they were assigned - do attempt to account for compliance behaviours, they rely on the assumption that the now non-randomised groups are comparable. This is unlikely to hold and may result in estimates of the treatment effect that are subject to selection bias [4].

Previous systematic reviews have identified statistical methods to deal with this issue in non-inferiority trials [6]​ and in time-to-event and health technology assessment (HTA) contexts [7]. Additionally, Mostazir et al. (2019) conducted a methodological review of RCTs in order to assess which methods are most commonly used to handle non-adherence to the protocol [8]. Methods identified across these reviews included principal stratification methods such as instrumental variables (IVs) and G-estimation methods such as marginal structural models (MSMs) with inverse probability of censoring or treatment weighting (IPCW/IPTW) and rank-preserving structural failure time models (RPSFTMs). Whilst these reviews provide a useful summary of the existing methods to deal with the issue of non-compliance in a range of contexts, they provide little information about the performance of these methods. Indeed, all three papers concluded that further work is required to assess and compare the performance of the methods that they identified [6–8]​.

Many of the papers identified in these reviews proposed new methods in order to address a specific scenario and evaluated their finite sample performance under simulation. Whilst using simulation in this manner is common practice, Boulesteix et al. (2013) argue that these papers should be treated with caution, since these simulations may be prone to “inventor bias” [9]. Pawel et al. (2022) also recently demonstrated how it is relatively easy to prove new methods to be optimal using simulation studies [10]​.

Applying the idea of the ‘phases of statistical methodology research’ framework recently proposed by Heinze et al. (2023), many of these papers could be described as covering a ‘phase I/II’ level of research. Heinze et al. noted that many methods are proposed without ever being fully investigated and introduced their framework in order to put more weight on studies that conduct carefully planned method comparisons which explore the empirical properties of methods in a wide range of scenarios [11]​.

A natural drawback of simulation studies is that, whilst they allow for precise simulation conditions relevant to the problem of interest to be specified, this may result in poor external validity. One potential solution to this issue is to conduct a systematic review of completed simulation studies. Collating and appraising the results from studies that have used simulations to assess existing methods in this manner would allow for those most widely considered to be compared and evaluated based on evidence from a number of studies. This would ensure consideration of a range of scenarios with some replication and improve overall inferences made about the area of research. This also combats any potential for misinterpretation of individual studies [12]​. Not only does this approach produce a summary of the performance of some key methods, it also provides a better picture of the landscape and progress of research in this area.

This systematic review aimed to identify all methodological papers that have evaluated and compared a number of existing methods to deal with non-compliance in RCTs using a simulation study. The results of this review could be used in order to identify gaps in current research, inform further work or provide guidance for applied researchers wanting to consider compliance to the intervention within their analysis. The goal of this review is to address the following questions:

1. Which methods to deal with non-compliance have been most thoroughly investigated by researchers undertaking simulation studies in this area and how do these methods perform under various scenarios?
2. What does this tell us about the research deficits in this area? (E.g., which methods need to be evaluated more rigorously?)

## Methods

This systematic review is reported using the most recent version of the PRISMA guidelines [13]. The PRISMA checklist is provided in the supplementary material 1 along with the review protocol. The review was listed on PROSPERO before it commenced (registration number CRD42022370910).

### Classification of methods

**Fig. 1:**
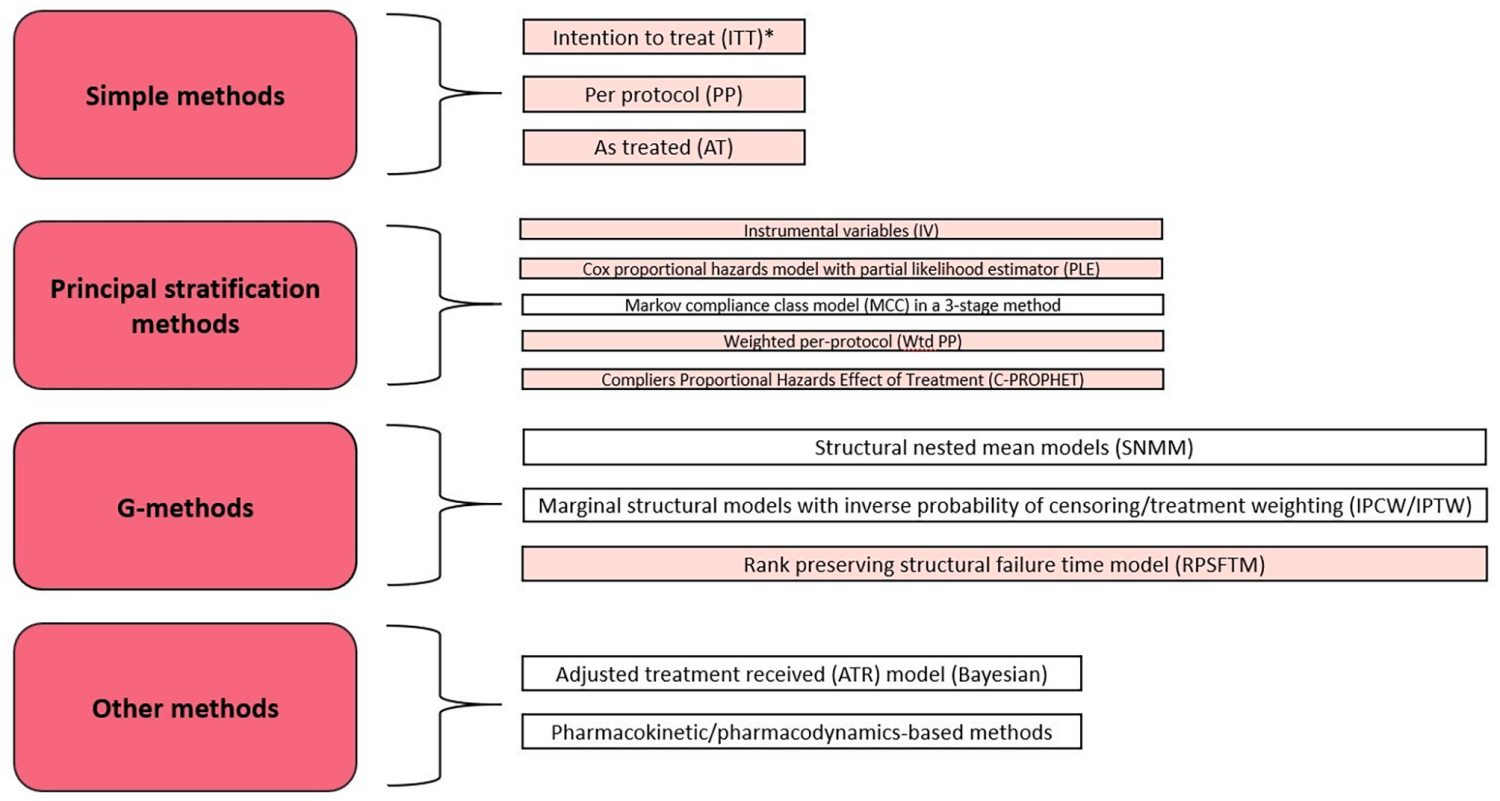
Taxonomy of methods to deal with non-compliance to the protocol in a RCT, adapted from the taxonomies of Alshreef et al. and Mostazir et al. [7, 8]. Methods were categorised as simple, principal stratification, G-methods or “Other”. Methods highlighted are ones that have been identified within papers included in this review. *ITT does not attempt to deal with non-compliance directly but is included here as a “do nothing” approach

Existing methods to deal with non-compliance in analysis of RCTs are summarised in Fig. 1. This summary was based on recent systematic reviews by Alshreef et al. (2019) and Mostazir et al. (2019) [7, 8] and is presented in order to provide an overview of methods that may be explored in the papers included in this review and highlight research gaps. Along with the results of this review, this will hopefully create a clearer picture of which methods have been explored in detailed simulation studies and which should be assessed further.

### Search strategy

A literature search was conducted in order to identify papers that focused on the comparison of existing methods to deal with non-compliance in RCTs using simulation. The online databases MEDLINE, Web of Science, Scopus and MathSciNet were searched using a combination of keywords from inception to 30th November 2022. These databases were selected with the assistance of a librarian specialising in medical and health information sources, in order to ensure all relevant papers were identified. MathSciNet was included in the case that any pertinent simulation studies happened to be absent from the medical literature. The original search strategy, which was developed for MEDLINE in PubMed and was adapted for the other databases, is available in the supplementary material 3. Ongoing studies were not included due to the methodological nature of this review. The reference lists of included papers were also searched by the primary author in order to identify any studies fitting the inclusion criteria that may have been missed in the database search.

### Inclusion and exclusion criteria

For a methodological paper to be included in this review, it must have been published in the English language in a peer-reviewed journal and focus on comparison of two or more existing methods to deal with non-compliance under a simulation study. Reasons for exclusion included focus on alternative issues in clinical trial analysis, development of a novel method or consideration of a specific setting other than a superiority RCT. A full list of inclusion and exclusion criteria are given in Table 1. An article must have satisfied all inclusion criteria in order to be included within the review. Note that the structure of the eligibility criteria given here differ slightly from that detailed in the protocol. This change was made in order to make these criteria clearer and to specify which area of the reviewed papers they correspond to. Additionally, one criterion has been removed, which excludes “theoretical papers with no application/assessment of method via simulation”. It was felt that this was covered within the other exclusion criteria and was therefore an unnecessary addition.

**Table 1:** List of inclusion and exclusion criteria for this systematic review. RCT - Randomised Controlled Trial

	Inclusion criteria	Exclusion criteria
Publication type	Peer-reviewed methodological papers whose focus is to compare two or more existing methods under a sufficient simulation study.	Non-peer reviewed articles, books or book chapters, theses or other grey literature such as conference proceedings.
Focus	The methodological topic of interest is non-compliance to the randomised intervention, which may be by participants in the intervention or control groups. This compliance could be described as all-or-nothing or time varying/partial. Some papers refer to non-adherence, but we are considering these terms to be interchangeable in this paper.	Papers that focus on issues such as missing data or the combination of these issues with non-compliance. Papers whose focus is a novel method rather than comparison of existing methods (e.g., that describe/reference a current method and propose an extension to it or propose a new method).
Setting	The methods considered are explicitly applied to account for non-compliance in the setting of a superiority RCT.	Papers that focus on an observational setting. Papers that consider a non-inferiority or equivalence setting. Methods based on aggregated data such as meta-analysis.
Simulation study	A simulation study was defined as ‘sufficient’ based on the following criteria: o The simulation study clearly states its objectives and gives a description of how the simulation was conducted/the nature of the simulated data. o The simulation study compares at least two existing methods that aim to account for non-compliance and estimate a point estimate of the intervention effect. o Existing methods refers to those that have not been proposed in the paper of interest and the authors have referenced previous work when describing the method. o The authors consider several non-compliance scenarios, such as varying the proportion or type of non-compliance. o Amongst performance measures, at least the bias of methods is reported or can be easily deduced.	
Publication date	Papers published from databases inception to 30th November 2022.	
Publication language	Papers published in the English language.	

Since it was important to ensure high quality of simulation studies, one of the inclusion criteria specified that only “sufficient” simulation studies should be included in the review. In the absence of a validated tool for assessing the risk of bias for simulation studies, the following criteria were used to define this, based on guidance for reporting simulation studies by Burton et al. (2006) and an overview of previously identified relevant papers [14] .

- The simulation study clearly states its objectives and gives a description of how the simulation was conducted/the nature of the simulated data.
- The simulation study compares at least two existing methods that aim to account for non-compliance and estimate a point estimate of the intervention effect.
- Existing methods refers to those that have not been proposed in the paper of interest and the authors have referenced previous work when describing the method.
- The authors consider several non-compliance scenarios, such as varying the proportion or type of non-compliance.
- Amongst performance measures, at least the bias of methods is reported or can be easily deduced.

### Screening

Search results were imported into the Covidence software [15] and duplicates removed. Title and abstract screening was conducted independently by two reviewers (L.A. and F.M.). Full-text screening was conducted with three reviewers (L.A., F.M. and A.C.J.), with each study being reviewed independently by L.A. and one other reviewer. Any conflicts were resolved by discussions including all reviewers until a consensus was reached.

### Data extraction and synthesis

A predetermined extraction form produced in Covidence was used for data extraction, which was piloted on a couple of studies by the primary author to ensure it was adequate. General information such as title, authors, year, journal and country were extracted. Additionally, outcomes of interest included the methods, trial setting and definition of non-compliance considered as well as details of the simulation study. These included the scenarios varied and the performance measures reported. Finally, the key findings and conclusions of authors were also extracted. In general, this information was extracted wholly to prevent misinterpretation.

Data extraction was performed independently by two of three reviewers (L.A., F.M. and A.C.J.) in the same manner as full-text screening, with differences resolved by consensus. Extracted information was exported and tabulated. Descriptive statistics, tables and graphs were used to explore and summarize the data and conclusions were drawn from these inferences.

A quality assessment form was used to assess the general quality of the papers included in the review and this information was summarised and reported. This assessed the reporting of the simulation study, whether there was any justification or discussion by authors of assumptions made throughout the simulation set-up, values used during data generation and the number of simulations. It also considered whether the conclusions made by the authors were supported by the results of their simulation study, whether the authors appeared to have any bias towards a particular method and the generalisability of their results. These criteria were constructed by the primary author, based on areas where it was thought that bias or ambiguity may be present, as well as simulation study reporting guidelines. For example, in certain papers it is clear that the authors are interested in one method in particular rather than an objective comparison of methods and this is an important consideration alongside the papers’ conclusions. Additionally, the settings considered within the simulation study may impact the generalisability of its results. This information gives greater context for the reader, which is an important aspect of any conclusions made.

## Results

**Fig. 2:**
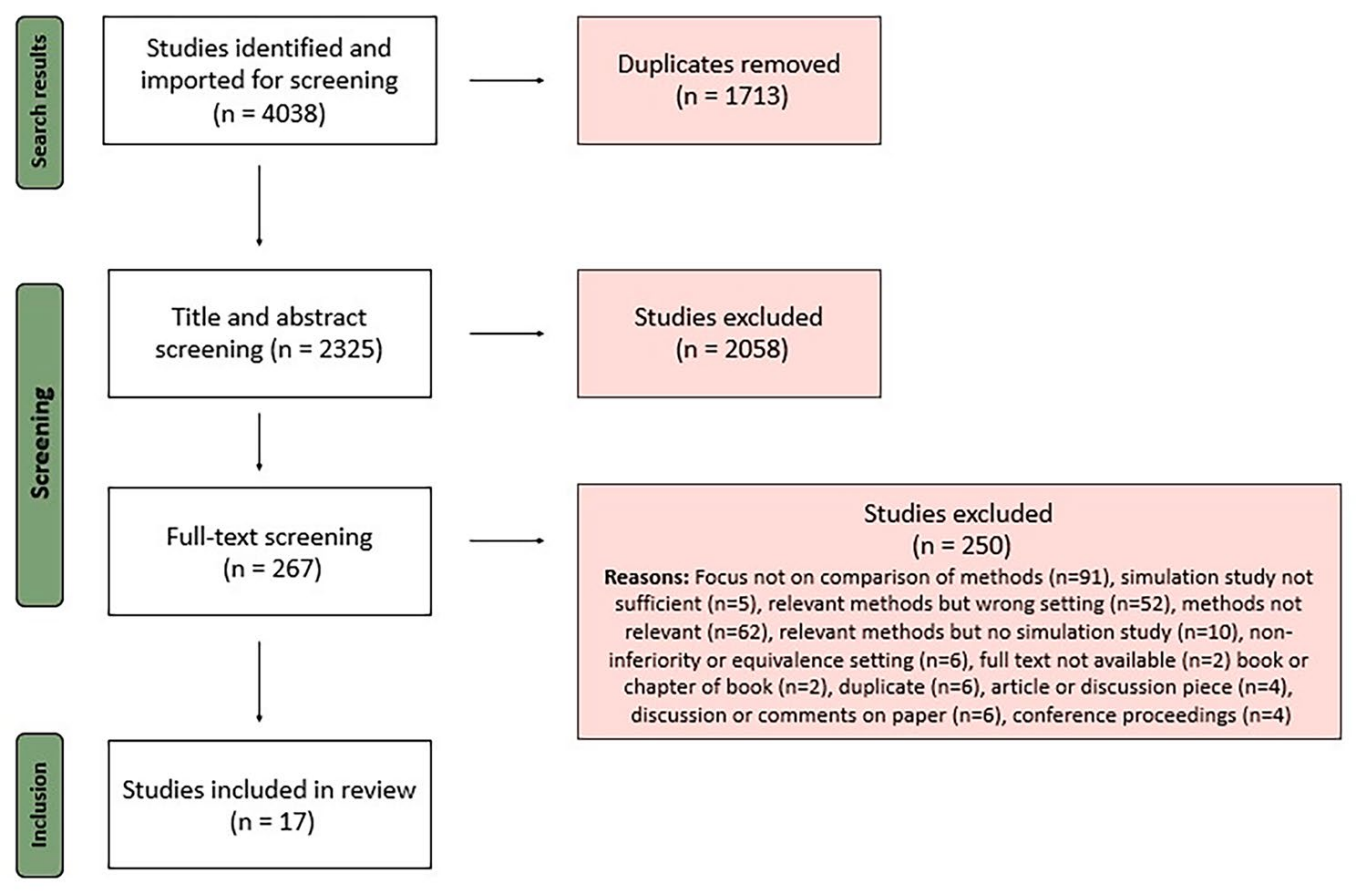
PRISMA flow diagram illustrating the screening process

Figure 2 shows the number of studies included in each stage of this systematic review. Initial searches returned 2325 studies for title and abstract screening once duplicates had been removed. We assessed 267 full texts, which resulted in 17 studies included in the final review. Five studies were excluded based on the quality and relevance of their simulation studies, which focused on power and coverage probabilities rather than a variety of performance measures, only varied sample size and no other factors or did not compare methods [16–20]. No further papers meeting the inclusion criteria were found during a search of the reference lists from the selected papers.

### Definition of non-compliance

**Fig. 3:**
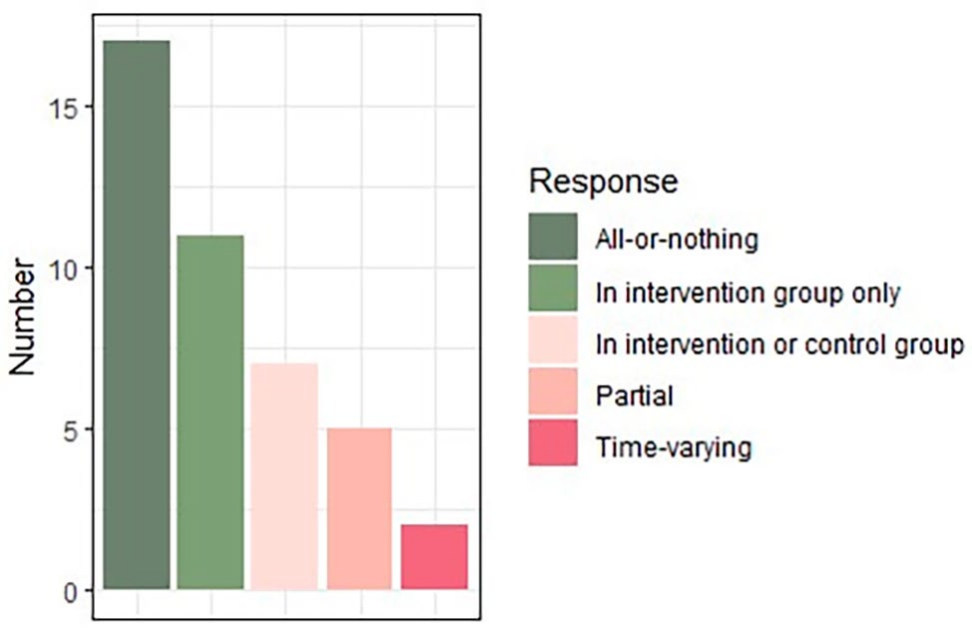
Frequency of different definitions of non-compliance given across studies

Figure 3 summarises the types of non-compliance considered by authors. Most non-compliance was assumed to be all-or-nothing, defined as a binary variable where individuals are supposed to either fully comply with the protocol or not comply at all (17 papers). Additionally, it was often implemented in the intervention group (11 papers), based on the monotonicity assumption often being made within the principal stratification framework. However, seven papers considered methods that allow non-compliance to be partial or time-varying and some authors considered both of these settings simultaneously, depending on the method applied during analysis (six papers).

### Estimand of interest

Five papers clearly specified that they were interested in estimation of the complier average causal effect (CACE) or the local average treatment effect (LATE) among compliers. The remaining twelve only referred to estimating the treatment effect and did not specify further, although some mentioned that the estimand of interest may differ between the methods considered.

### Methods considered

**Fig. 4:**
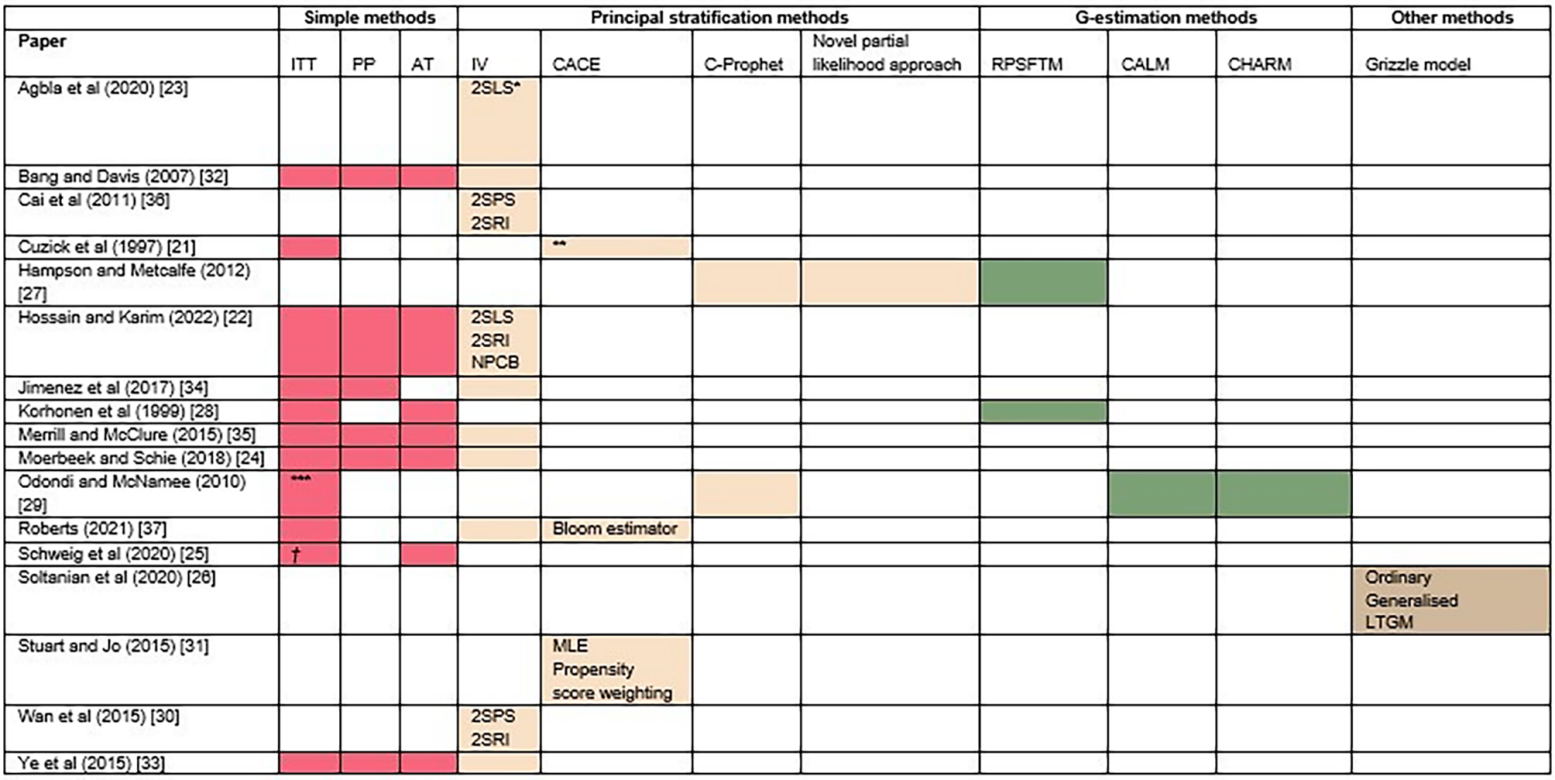
Methods included in each of the articles. Where estimators of the same method are compared, these are specified within the table. 2SPS – Two-stage predictor substitution; 2SRI – Two-stage residual inclusion; 2SLS – Two-stage least squares; NPCB – Non-parametric causal bound; MLE – Maximum likelihood estimation; RCT – Randomised controlled trial; IV – Instrumental variables; ITT – Intention to treat; AT – As treated; PP – Per protocol; RPSFTM – Rank preserving structural failure time model; CALM -Causal accelerated life model; C-Prophet - Compliers proportional hazards effect of treatment; CHARM - Causal hazard ratio adjustment regression model; CACE – Complier average causal effect; LTGM – Latent treat grizzle model; *Compare ordinary and weighted least squares methods of adjustment for CL variable. **Compares ITT to unnamed “corrected method”. ***Also compare Cox model with binary and time-varying covariate. *†*Compares HLM with as-assigned or as-treated cluster

Figure 4 shows the methods considered and compared in each paper included within the review. These have been grouped into categories in a similar manner to the taxonomy presented in Fig. 1, but where specific estimators of a method have been compared, these have been noted within the table. Where a method included within this taxonomy appeared within a paper included in this review, it has been highlighted in order to emphasize this.

Table 2 provides general information about the papers included in this review. Nine papers (53%) were from Statistics in Medicine and two from Statistical Methods in Medical Research. Others came from various other journals. The majority of papers (82%) had been published since 2010, with the oldest from 1997 [21] and the most recent from 2022 [22]. In general, the key aims of the papers included focused on comparison of methods or estimators, as stipulated by the inclusion criteria, but some focused on specific settings such as cluster randomised trials [23–25], cross-over trials [26] or time-to-event data [27–30] and considered issues such as inclusion of baseline information and the impact of unmeasured confounding.

**Table 2:** Title, author, year, journal and a summary of objectives of the papers included in this review. 2SPS – Two-stage predictor substitution; 2SRI – Two-stage residual inclusion; 2SLS – Two-stage least squares; RCT – Randomised controlled trial; IV – Instrumental variables; ITT – Intention to treat; AT – As treated; PP – Per protocol; CALM – Causal accelerated life model; C-Prophet - Compliers proportional hazards effect of treatment; CHARM - Causal hazard ratio adjustment regression model; CACE – Complier average causal effect; LTGM – Latent treat grizzle model; HR – Hazard ratio

Author(s)	Publication Year	Title	Journal	Main aims and objectives
Agbla et al. [23]	2020	Estimating cluster-level average treatment effect in cluster randomised trials with non-adherence	Statistical Methods in Medical Research	o Comparing alternative estimation strategies for the implementation of 2SLS estimation using cluster-level data. o Demonstrate that using individual-level covariate-adjusted cluster summaries in the (weighted) 2SLS regression can increase efficiency.
Bang and Davis [31]	2007	On estimating treatment effects under non-compliance in randomized clinical trials: Are intent-to-treat or instrumental variables analyses perfect solutions?	Statistics in Medicine	o Compare the performance of four estimators that are conventionally considered for treatment effect estimation under different non-compliance scenarios in a typical clinical trial setting under simulation.
Cai et al. [32]	2011	Two-stage instrumental variable methods for estimating the causal odds ratio: Analysis of bias	Statistics in Medicine	o Present analytical and simulation results for the bias of 2SPS and 2SRI estimators under a causal logistic model expressed in terms of potential outcomes under the principal stratification framework.
Cuzick et al. [21]	1997	Adjusting for non-compliance and contamination in randomized clinical trials	Statistics in Medicine	o Study a method of analysis which estimates the magnitude of the treatment effect among compliers in a randomized study in such a way as to respect the randomization and still be valid even when compliers have a different baseline risk than non-compliers.
Hampson and Metcalfe [27]	2012	Incorporating prognostic factors into causal estimators: A comparison of methods for randomised controlled trials with a time-to-event outcome	Statistics in Medicine	o Discusses the problem of making causal inferences in trials with a survival outcome when a proportion of patients allocated to the active intervention do not receive it, and prognosis in the absence of the intervention differs between those who comply and do not comply. o Focuses on the case where treatment switches occur at baseline. o Compares three estimators of the causal effect of treatment on compliers using simulated data.
Hossain and Karim [22]	2022	Analysis approaches to address treatment nonadherence in pragmatic trials with point-treatment settings: a simulation study	BMC Medical Research Methodology	o Compare the performance of four methods to address non-adherence; two adjusted PP approaches and three versions of the IV-based method in the presence of nonadherence. o Identify which methods are more appropriate to use under the scenarios where their respective assumptions are violated.
Jimenez et al. [33]	2017	Evaluating the effects of treatment switching with randomization as an instrumental variable in a randomized controlled trial.	Communications in Statistics – Simulation and Computation	o Utilises simulated data based on an ongoing RCT to evaluate the effects of treatment switching with randomisation as an instrumental variable at differing levels of treatment crossovers, for continuous and binary outcomes. o Data were analysed using IV, ITT and PP methods.
Korhonen et al. [28]	1999	Correcting for non-compliance in randomized trials: An application to the ATBC study	Statistics in Medicine	o We compare the performance of the ITT, AT and g-estimation approaches under different setting for non-compliance with emphasis on the case where there are unmeasured confounders at baseline affecting both treatment-free survival time and time on active treatment.
Merrill and McClure [34]	2015	Dichotomizing partial compliance and increased participant burden in factorial designs: the performance of four noncompliance methods	Trials	o Using simulations, we assessed the performance of ITT, PP, AT and IV in both the partial compliance setting and in a 2-by-2 factorial design with increased participant burden for those randomised to both active treatments.
Moerbeek and Schie [24]	2018	What are the statistical implications of treatment non-compliance in cluster randomized trials: A simulation study	Statistics in Medicine	o Investigate the statistical implications of non-compliance in cluster randomized trials. o A simulation study was conducted with varying degrees of non-compliance at either the cluster level or subject level, which compared ITT, AT, PP and IV methods.
Odondi and McNamee [29]	2010	Performance of statistical methods for analysing survival data in the presence of non-random compliance	Statistics in Medicine	o Compares methods (CALM, C-Prophet, CHARM, Cox-Reg1, Cox-Reg2) and the ITT (Cox-ITT) method when applied to trials where there is time-dependent unidirectional noncompliance in the active arm. o A principal objective is to compare performance of methods which treat compliance as binary (Cox-Reg1 and C-Prophet) and those which utilize the time when subjects switch to noncompliance (Cox-Reg2, CALM and CHARM).
Roberts [35]	2021	The implications of noncompliance for randomized trials with partial nesting due to treatment group	Statistics in Medicine	o Considers the following questions in the setting of nested clustering, whereby clustering only exists in the intervention arm: 1. How do methods for estimating ITT effects using intended group/actual group perform? 2. Where both are recorded, which should be used for estimating ITT effects? 3. How do methods for estimating the CACE perform?
Schweig et al. [25]	2020	Switching Cluster Membership in Cluster Randomized Control Trials: Implications for Design and Analysis	Psychological Methods	o With a focus on cluster switching that violates treatment assignment, goal of article is to explore the challenges posed for analysis of clustered RCTs and propose a potential solution to these challenges. o Address three research questions using real data as well as a series of Monte Carlo simulations: (a) To what extent can inferences about program effects differ when using as-treated or as-assigned clusters? (b) Under what conditions are choices about modelling clustering consequential? Does it depend on the extend of noncompliance or assumptions about the source(s) of between-cluster variability? (c) Are any approaches preferable to others?
Soltanian et al. [26]	2020	Analysis of crossover clinical trial in the presence of non-compliance: a two-stage latent treat grizzle model	JP Journal of Biostatistics	o Compare the accuracy of three models: ordinary grizzle model, generalised grizzle model and LTGM model under different simulated scenarios. o In this article, have tried to use the effect of baseline variables on patients’ compliance and estimate the treatment effects by maximising the likelihood function.
Stuart and Jo [36]	2015	Assessing the sensitivity of methods for estimating principal causal effects	Statistical Methods in Medical Research	o Discuss and examine two methods that rely on very different assumptions to estimate the CACE. o Details the assumptions underlying each approach, and assess each method’s sensitivity to both its assumptions, and those of the other method using both simulated data and a motivating example.
Wan et al. [30]	2015	Bias in estimating the causal hazard ratio when using two-stage instrumental variable methods	Statistics in Medicine	o Directly compare bias in causal HR estimated by 2SRI and 2SPS using extensive simulations.
Ye et al. [37]	2014	Estimating treatment effects in randomised controlled trials with non-compliance: a simulation study	BMJ Open	o Through simulation, we aim to compare common approaches in analysing non-compliant data under different non-compliant scenarios. o Objectives were to compare the performance of these different approaches and make recommendations on optimal approaches under specific scenarios.

Five papers compared ITT, PP, AT and IV analysis approaches, with a number also focused on comparison of different IV estimators (4 papers, 24%). Others also looked at estimators of the CACE or comparison of a range of other methods, including G-estimation methods, although this was less common. Aside from this, two papers were more unique in the methods they considered. Cuzick et al. (1997) compared the ITT approach to a ‘corrected method’, which utilises the principal stratification framework and further extended this model to allow for time factors, developing a time-stratified constant relative risk model, although these methods are not named [21]. Additionally, Soltanian et al. (2020) focused on the Grizzle model, which addressed non-compliance in crossover trials, and compared the ordinary and generalised versions of it to the latent-treat non-compliance model [26]. As well as comparison of different methods and estimators, it was also common for authors to consider different forms of adjustment, calculation of robust standard errors (SEs) and incorporations of baseline covariates.

### Simulation study

All simulation studies varied compliance scenarios in some way, since this was a criterion for inclusion in the review (Table 3). Across papers, it was common for simulation studies to vary the rate or levels of non-compliance, as well as its dependence on other factors. For example, some assumed non-compliance to be random, whereas others considered it to be related to the outcome or other measured or unmeasured confounders. Some papers also looked at different types of non-compliance within the simulation scenarios, such as all-or-nothing or partial compliance and changed whether it was possible to be non-compliant in the control group or just the intervention group, whereas others specified this earlier in the paper and did not consider alternatives during simulation.

**Table 3:** Summary of the simulation study or studies conducted in each paper, including the key findings and conclusions of the authors. ICC – Intra-cluster correlation coefficient; LATE – Local average treatment effect; SE – Standard error; SSDF – Small sample degrees of freedom; CI – Confidence interval; 2SLS/TSLS – Two-stage least squares; CL – Cluster level; CP – Coverage probability; ITT – Intention to treat; PP – Per protocol; AT – As treated; IV – Instrumental variable; MSE – Mean squared error; 2SRI – Two-stage residual inclusion; 2SPS – Two-stage predictor substitution; IP-weighted – Inverse probability weighted; ER – Exclusion restriction; OR – Odds ratio; RCT – Randomised controlled trial; RMSE – Root mean squared error; C-Prophet - Compliers proportional hazards effect of treatment; CALM – Causal accelerated life model; OLS – Ordinary least squares; GEE – Generalised estimating equations; HLM – Hierarchical linear model; SD – Standard deviation; LTGM – Latent treat grizzle model; ER – Exclusion restriction; HR – Hazard ratio

Paper	Compliance scenarios varied	Other scenarios varied	Performance measures	Key results
Agbla et al. (2020) [23]	o Non-compliance at either cluster or individual level o Expected probability of compliance differs between these o Effect of individual and cluster level variables on odds of adherence is varied	o Vary number of clusters, average cluster size, ICC, strength of confounding and true value of LATE o Effect of individual and cluster level variables on outcome is varied o Within analysis, consider different methods for weighting, SE estimation and SSDF correction	o Empirical bias + Monte Carlo SE o Coverage rates of 95% CIs	o Shows that TSLS regression applied to CL summaries is a simple, valid method for obtaining LATE estimates. o All weighting strategies perform similarly when number of clusters is not small. o Minimum-variance weights generally perform well unless there are very few clusters or outcome ICC is large. o Cluster-size weights should not be used when cluster sizes are variable. o Authors give a useful table of recommendations for different adherence scenarios.
Bang and Davis (2007) [31]	o Non-compliance scenarios considered - Can occur in either treatment arm - Only in intervention arm - Partial compliance o Within each of these, also varied whether non-compliance was ignorable or symmetric/asymmetric	o Considered two different true treatment effects	o Mean o Sum of squared errors o Coverage probability	o IV estimator behaves best and improves upon ITT in terms of bias and CP. o However, bias of IV not always negligible and IV can be as problematic as PP and AT depending on underlying scenario, except in the hypothetical setting of a constant treatment effect. o Identify a trade-off between increased information and more reliable statistical properties, since IV requires additional, accurate information and verification of underlying assumptions, which the ITT does not.
Cai et al. (2011) [32]	o Varied probabilities of being an always-taker, complier of never-taker	o Magnitude of confounding is also varied	o Observed bias o MSE	o Confirm results of previous papers, which show that 2SRI estimator is unbiased when true model is conditional on unmeasured confounder and that the 2SRI bias increases as the magnitude of confounding increases for the treatment effect conditional on compliance. o Similar results hold for the 2SPS estimator, except that 2SPS is biased even when there is no unmeasured confounding. o This bias occurs even when all IV assumptions are met.
Cuzick et al. (1997) [21]	o Varied rate of non-compliance and contamination	o Varied benefit of treatment, randomisation ratio and total trial population	o Bias o Confidence intervals	o “Corrected method” produces larger treatment effects than ITT when baseline failure rates in non-compliers and contaminators are the same as those who accept their allocated treatment and confidence limits are also wider. o “Corrected method” provides a better estimate of the true treatment effect and more realistic confidence intervals.
Hampson and Metcalfe (2012) [27]	o Proportion of noncompliers varied o Varied whether effects of compliance on hazard of mortality were strong or weak	o Considered whether compliance indicator and important baseline covariates were correlated or independent o Also considered models both adjusted and unadjusted for baseline covariates	o Mean o Percentage bias o Coverage of 95% CI o Power	o Methods of estimating causal treatment effects for time-to-event outcomes can be extended to incorporate covariates. o All three methods are accurate when an important covariate was included in the model, with a maximum bias of 5.4%. o When there are strong prognostic factors, it is important to adjust efficacy estimates for them in order to avoid biased, whether or not these factors are associated with compliance. o Generally, it is hard to regain power for testing causal treatment effects, no matter how sophisticated the method of analysis.
Hossain and Karim (2022) [22]	o Varied nonadherence rate	o Considered weak and strong confounding, null and non-null effect and minor or severe violation of the exclusion-restriction assumption.	o Bias o SE o MSE o 95% confidence interval probability	o No single method is the best in all situations. o Both-stage adjusted 2SLS and 2SRI perform well in terms of bias and coverage when known confounders are adjusted for and this has improved precision over the naïve approach. o IP-weighted PP outperforms these approaches in terms of bias, SE and MSE for < 80% nonadherence but shows high bias for nonadherence greater than this and does not perform so well when there is unmeasured confounding. o All methods can have bias when the ER assumption is violated. However, baseline-adjusted PP and IP-weighted PP can be unbiased if all open backdoor paths between the treatment variable and the outcome can be blocked.
Jimenez et al. (2017) [33]	o Level of treatment switching/noncompliance varied o Crossover considered from both treatment groups o Compliance considered as both random and based on diagnosis	o Varied risk score effect size, OR for death and OR for high coronary artery disease risk	o Bias o Root MSE o CI coverage probability o Empirical power	o PP analysis can provide biased model estimates when non-compliance is not random. o ITT analysis generally gives more biased estimates with lower coverage probabilities and lower power in some cases compared to IV as levels of treatment group switching increase. o IV performed better than ITT in most cases where there was a treatment effect but ITT was slightly better in the null case, although IV was just as good at low levels of switching. o IV can have higher model estimate variance and greater CI widths as rate of switching increases, which is a trade-off for accurately estimating a true treatment effect whilst preserving a RCTs randomisation.
Korhonen et al. (1999) [28]	o Varied non-compliance rate and whether it is dependent on outcome or not	o Varied treatment effect o Treatment-free survival considered as both dependent and independent of time on active treatment	o MSE o Coverage of 95% CI o Power o Bias	o ITT analysis often gives estimates that are biased towards the null but is valid for testing purposes, as provided the study has sufficient power the ITT would reject the null hypothesis if a true treatment effect existed. o AT approach can be misleading when non-compliance is outcome dependent. o G-estimation provides valid estimates when the underlying structural model is correct, even when non-compliance is outcome dependent. However, it introduces extra censoring and hence, a loss of power is induced.
Merrill and McClure (2015) [34]	o Range of different noncompliance scenarios considered using different distributions (beta and uniform) o Allowed compliance to be both independent and not independent of other factors o Range of cutoff points considered since partial compliance was dichotomized	o Considered both a two-arm trial design and a factorial design o Considered null and true treatment effect	o Average bias o MSE o Power	o Use of PP and AT provides little benefit over ITT when compliance is dichotomized, whilst use of IV in this case often led to unacceptably inflated type I error rate. o This may also be the case for PP and AT, especially if the compliance distribution does not cluster around 0 or 1. o Results for factorial design similar to two-arm trial. Increased burden for participants mainly affected results through increased levels of overall non-compliance in study population.
Moerbeek and Schie (2018) [24]	o Level of non-compliance varied o Non-compliance considered at the subject and cluster level	o ICC, cluster size and number of clusters varied o Each data set analysed with and without a covariate effect	o Mean estimate compared to true effect (bias) o Standard deviation o Coverage o Power o Partial F statistics for IV method	o Non-compliance may result in severely biased results. o AT and PP may underestimate population value of target estimand when covariate not included in model, and this becomes more severe as the probability of non-compliance increases. o Standard errors of AT, PP and IV increase with level of non-compliance. o In general, results get worse when probability of non-compliance increases and when covariate that influences compliance is not included in statistical model. o Conclude that avoiding non-compliance is best but where this is not possible, covariates related to compliance should be included in the statistical model.
Odondi and McNamee (2010) [29]	o Non-compliance considered to be both random and non-random o Correlation between non-compliance and hazard (how much it depends on a patient’s condition) also varied	o Considered two different treatment effects	o Bias o SE o RMSE o 95% CI coverage	o While the time-dependent method is adequate under random compliance it breaks down under non-random compliance with the bias related to the magnitude and direction of correlation between risk and probability of non-compliance. o All specialist methods performed well in terms of bias, even C-Prophet which took compliance as all-or-nothing but coverage of this method was low. o CALM performed best in terms of bias and coverage but had largest RMSE. o G-methods may be more valuable in general as can be extended to explore lagged treatment effects, for example.
Roberts (2021) [35]	o Consider different values of the ratio of variance of compliers in the intervention arm and never-takers in the control arm o Also vary the difference between control compliers and never-takers and the compliance rate	o Vary ICC and group size	o Bias o Coverage	o ITT estimates based on random effects model or GEE with exchangeable correlation matrix performed better when using intended group over actual group. o OLS with robust SEs performed well with both intended and actual group. o Most CACE models performed well. o Conclude that it is desirable to record both intended and actual group analyses, as ITT with mixed models can be fitted using intended group with data generation assumptions checked by a causal model using actual group. o When ITT based on actual group, a worse outcome for never-takers over compliers may allow one to infer that some estimators are biased towards the null treatment effect. o Generally, the weighting of data by the method of analysis may induce bias where the outcome of subjects in clusters differs from those that are not.
Schweig et al. (2020) [25]	o Level of non-compliance o Consider 2 conditions with non-compliance just in the intervention group and 1 with it in both intervention and control groups	o Number of clusters and ICC varied o Also looked at different values for the proportion of ICC that was attributable to provider effects	o Relative bias o Relative bias in SEs	o Using the AT cluster in HLM will bias the ITT estimate and using as-assigned cluster will bias the standard error estimates when heterogeneity among clusters is due to heterogeneity in the treatment effects. o Using OLS/linear regression with two-way cluster adjusted SEs can yield unbiased ITT estimates and consistent SEs regardless of the source of random effects and recommends this method to replace HLM in the setting of non-compliance and cluster switching.
Soltanian et al. (2020) [26]	o Three non-compliance rates considered	o Three sample sizes considered	o Mean – average treatment effect o SD of simulated estimates o Empirical bias of simulated estimates	o Simulation study showed that the LTGM model has the lowest bias in all cases.
Stuart and Jo (2015) [36]	o Vary the strength of the relationship between a covariate and compliance	o Consider violation of three key method assumptions: - Exclusion restriction - Normality - Principal ignorability	o Bias o Empirical SE o RMSE o Coverage (95%)	o ER based joint approach appears less sensitive to assumptions. o Performance of both methods is significantly improved when there are strong predictors of compliance. o Interestingly, both methods perform particularly well when the assumptions of the other are violated, highlighting the importance of carefully selecting an estimation procedure.
Wan et al. (2015) [30]	o Vary the “strength of confounding”/non-compliance o Probability of being an always taker and complier set to three combinations, representing low, medium and high levels of compliance.	o Vary the hazard rate o Vary the magnitude of unmeasured confounding o Probability of being assigned to treatment set to 0.1 or 0.5 to reflect both new and relatively established treatments.	o Bias o MSE	o 2SRI and 2SPS approaches are both biased in estimating the causal HR among compliers, especially when hazard is increasing, even under a moderate amount of unmeasured confounding. o 2SPS less biased when hazard is decreasing. o Even when all assumptions are met, both methods could fail to consistently estimate causal HR. o Recommend exercising caution when interpreting results from two-stage IV survival models. o Analytic results for bias may help guide researchers in deciding when two-stage IV methods may be reasonably applied.
Ye et al. (2015) [37]	o Vary the type, randomness and degree of non-compliance		o Bias o MSE o 95% coverage	o Standard ITT is biased under non-compliance when the intervention has a moderate or large effect, but is the optimal approach when estimating a null effect. o When patients’ non-compliance behaviour was random, the AT, PP, IV and CACE approaches all provided unbiased estimates. For other scenarios, the optimal method varied. o The authors provide a useful figure to help researchers choose the best method based on the scenarios considered in this paper.

Other quantities that were varied during simulations generally depended on the clinical trial setting and the aims of the paper. For example, papers looking at the relationship between non-compliance and clustering varied the number of clusters, average cluster size and intra-cluster correlation coefficient (ICC) and also considered the impact of cluster-level variables on the outcome. It was common for authors to consider different sample sizes and true effect sizes, as well as the strength of confounding or effect of baseline covariates. Finally, one paper also considered the impact of key methodological assumptions and whether they were violated or not [36].

The most common performance measures were bias (17 papers, 100%), 95% coverage probabilities (12 papers, 71%) and mean squared error/restricted mean squared error (MSE/RMSE) (9 papers, 53%). SE and empirical power were also reported in some cases (6 papers, 35% and 5 papers, 29% respectively). Monte Carlo standard error (MCSE), sum of squared errors (SSE) and the partial F statistic assessing the strength of the instrument for the IV method were given in one paper each [23, 24, 31].

### Findings

Six of the 17 papers compared principal stratification methods such as IV and CACE methods to ITT, PP and AT. Ye et al. (2015) found that IV was unbiased when non-compliance was random [37], although Bang and Davis (2007) concluded that IV may be as problematic as AT and PP in other scenarios, suggesting a trade-off between increased information and more reliable statistical properties [31]. Jimenez et al. (2017) similarly concluded that IV performed well in terms of bias when there was not a null treatment effect, but can have a higher variance and greater confidence interval (CI) widths, also proposing a trade-off between accurate estimation of the treatment effect whilst preserving randomisation [33]. Hossain and Karim (2022) concluded that no method was best in all scenarios, whilst considering a number of IV estimators as well as ITT, PP and AT, and that the optimal method was dependent on the setting and model assumptions [22]. Additionally, Merrill and McClure (2015) found that IVs lead to inflated type I error when partial compliance was dichotomized, which is common in practice [34].

Looking at the comparison of specific IV estimators, which is the focus of four of the papers in this review, Wan et al. (2015) found that two-stage predictor substitution (2SPS) and two-stage residual inclusion (2SRI) methods were both biased when there was an increasing hazard, and therefore, researchers should exercise caution when implementing these methods [30]. Cai et al. (2011) compared the same estimators and found 2SRI to perform better generally, but also reported that it was still biased when there was unmeasured confounding [32]. Agbla et al. (2020) alternatively compared weighting strategies applied to the two-stage least squares (2SLS) method of estimation in a cluster setting and concluded that 2SLS is a valid method and that all weighting strategies perform well, provided the number of clusters is not small [23]. Finally, Stuart and Jo (2015) compared a propensity score weighting approach to an exclusion restriction (ER) joint maximum likelihood estimation (MLE) method under a simulation study that considered violation of methodological assumptions and found the latter to be less sensitive to these conditions [36].

Only three papers considered G-estimation methods. One concluded that G-estimation provides valid estimates over ITT and AT but induces loss of power due to extra censoring [28]. Odondi and McNamee (2010) compared a wide range of methods, but also found the G-methods to be most valuable, especially the causal accelerated life model (CALM), which performed best in terms of bias and coverage. They also found that the compliers proportional hazards effect of treatment (C-Prophet) method performed surprisingly well in terms of bias, even though it forces a dichotomy on partial compliance, although it did have low coverage [29]. Hampson and Metcalfe (2012) found that the C-Prophet, Novel partial likelihood and RPSFTM methods were accurate when important covariates were included in the model and hence, advised that these should be adjusted for [27]. Similarly, Moerbeek and Schie (2018), who focused on the relationship between clustering and non-compliance, also advised that any covariates related to compliance should be included in the statistical model [24].

### Quality assessment

**Fig. 5:**
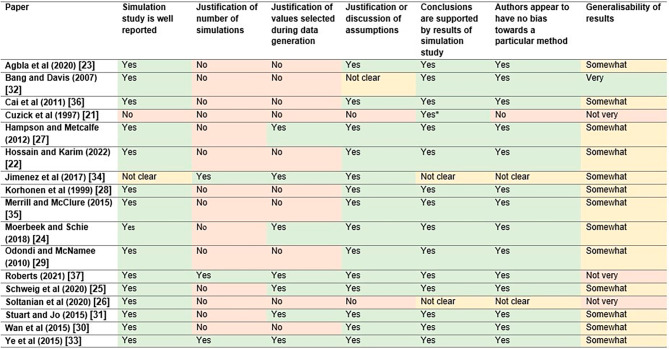
Quality assessment of the papers included in this review. *Paper contains no official discussion or conclusion section, but makes some conclusions in their abstract

Generally, simulation studies were well reported, with the majority of authors justifying or discussing any assumptions that they made (Fig. 5). However, less than half justified all values selected during data generation and only three papers gave a justification for the number of simulations run [33, 35, 37].

The conclusions of most papers were deemed to be supported by the results of the simulation study, although for two it was judged that this was unclear [26, 33] and these papers were also the ones that appeared to have potential bias towards or favour a particular method. The results from the majority of papers were deemed “somewhat generalisable”, with their general applicability predominantly being limited by the specificity of the settings that they were based upon or the scenarios that they considered. Three papers were judged as “not very generalisable”, either for this same reason or for previous issues mentioned with their quality [21, 26, 35], whilst one was deemed to be “very generalisable” [31].

Based on these results, it appears that the quality of papers included in this review is generally good, although in some cases better justification could have been given for the specifics within the setup of simulation studies. The conclusions of certain papers should perhaps be taken with caution and for this reason, less focus has been put on them when formulating the conclusions of this review.

## Discussion

This systematic review has shown that it is common for simulation studies assessing methods to deal with non-compliance to consider IV methods, either comparing these to ITT, AT and PP approaches or comparing different IV estimators. However, whilst is appears that IVs may be a popular method, many authors found the approach to be biased in several circumstances and this finding holds for a range of estimators.

Another key finding was a lack of comparison of methods that allow for time-varying non-compliance (G-methods). From assessment of the literature excluded in this review, it is clear that these methods are more commonly assessed in relation to treatment switching. Treatment switching often occurs in cancer trials and refers to the scenario where patients in the control arm are permitted onto the treatment arm at some point during follow-up, such as disease progression [38]. This differs from the type of non-compliance considered within this paper, primarily since treatment switching is usually pre-specified in the protocol and hence, is not a protocol deviation. A list of papers that compare relevant methods in a treatment switching setting is provided in the supplementary material 2. These papers were not included in this review due to the specific nature of the setting considered within their simulation studies (only participants in the control group were able to switch onto the intervention arm). However, it is important to note that there is a lot of overlap between these issues within the field of causal inference.

Only one paper identified within this review considered a wide range of different methods, comparing simple methods, principal stratification methods and G-methods. This paper found the CALM to perform best in terms of bias and coverage across the settings considered and that the C-Prophet method performed well in terms of bias but had low coverage. It would be useful for more simulation studies to be conducted that compare a range of different types of methods in this manner. This would ensure that there is a strong body of evidence on the performance of methods which account for non-compliance in a range of settings, including replication of results across independent simulation studies.

Previous systematic reviews in this area have identified methods to deal with non-compliance in various contexts, but all concluded that additional work was necessary in order to compare these methods [6–8]. Therefore, the systematic review reported in this paper is unique from these other reviews, since it aimed to determine the scope of literature that focuses on comparing these methods and hence, identified papers conducting simulation studies in order to achieve this. Seventeen papers were found that fit these criteria, which is perhaps a surprisingly low number, given the amount of literature on the methodological issue of non-compliance and how to deal with it. We found that many of the methods identified in these previous reviews have been examined under simulation, although certain G-methods such as MSMs with IPCW/IPTW and structural nested mean models were absent. Additionally, no independent assessment of Bayesian approaches was identified.

Whilst the intention of this systematic review was to summarise the current body of evidence and make recommendations for future work, rather than providing practical methodology-related guidance, the conclusions of authors are specific to the setting which they consider within their simulation studies and hence, their generalisability is limited by this. Additionally, it is important to remember that, in practice, the suitability of each causal method will be dependent on the clinical trial setting and the assumptions that the method makes. For example, the IV approach assumes that treatment allocation is not related to the outcome, other than via the risk factor of interest (the ER assumption) [4]. This may not hold for certain interventions, especially in trials in which blinding is not possible.

It is clear that further work is needed in this area. This should include additional simulation studies comparing a wide range of methods and specifically including G-methods, to provide a greater foundation of evidence of simulation results from which to base practical application. However, whilst the results of simulation studies are undoubtedly relevant and useful, it is important to remember that the ultimate aim is to improve the use of these methods within clinical trials. As Mostazir et al. (2019) found in their review of RCTs, a large proportion of trials still do not implement causal methods, such as the ones discussed in this paper [8]. This may be due to a lack of understanding of the counterfactual approach and the difficulty in explaining and implementing it. Therefore, a comprehensive overview of relevant methods and their advantages, limitations and assumptions would be beneficial to applied statisticians and those working in clinical trials.

This systematic review was planned with careful consideration and conducted in a structured manner, with two independent reviewers used to screen papers at all levels and conduct data extraction and quality assessment. In addition, best practice has been ensured by reporting using the PRISMA guidelines for systematic reviews. A recent scoping review of simulation studies comparing statistical and machine learning approaches to risk prediction for time-to-event data found that simulation studies often favour the method that was proposed within the paper itself and recommended that future comparison studies are conducted independently of developing a new method [39]. An attempt was made to minimise this type of bias within this review, by excluding papers that proposed a novel method and then considered it within their simulation study. In addition, this criterion was also included as part of the quality assessment. Only a couple of the papers included in this review were dubious in this sense, but this was predominantly because it was difficult to tell whether one of the methods considered had been proposed by the authors themselves.

A key limitation of this paper is the subjective nature of the criteria given for defining both a “sufficient” simulation study and assessing the quality of the studies included in the review, and the potential biases that this may introduce. The “sufficient” simulation study criteria were based on best practice reporting guidelines for simulation studies and were included with the aim of identifying only relevant simulation studies that were well reported in order to improve consistency in data extraction. No formal tool for assessing the risk of bias in simulation studies was identified and other similar reviews mentioned in this paper did not seem to have a formal quality assessment to take inspiration from. However, the authors felt that more bias would be introduced by failing to evaluate and report the risk of bias within each paper included in the review than by having an unvalidated quality assessment form. Therefore, a list of criteria was produced by L.A., based areas where it was deemed that bias may occur and simulation study reporting guidelines. These criteria were piloted on a few studies and peer reviewed by L.J.G. before being finalised.

A relative subjectivity was also necessary during data extraction and quality assessment, due to the narrative nature of the review. Overall, the authors aimed to be as objective as possible, using guidelines for reporting simulation studies to help assess the quality of them and focussing on summarising the conclusions of the original authors without changing their own interpretation of the results. Additionally, data extraction and quality assessment were conducted by two independent reviewers and any conflicts were discussed in depth. Finally, it is important to note that departures from the original protocol may induce a certain bias. In order to minimise this, deviations were only taken where essential in order to improve clarity in reporting the methodology of this review and all such deviations have been discussed and justified.

Systematic reviews of simulation studies are rarely conducted. One reason for this may be due to the difficulty in comparing results that are not equivalent. The lack of regularity in simulation study reporting means that summarising results and conclusions is complex and ambiguous. This undoubtedly impacts the generalisability of conclusions, despite the attempt made in this review to report a wide range of information about each included paper, such that the reader is able to make their own judgements where possible. Additionally, a recent systematic review on the quality of reporting of simulation studies about methods for the analysis of complex longitudinal patient-reported outcome data found that current reporting practices are not consistent with best-practice guidelines [40]. Recently published guidelines have aimed to provide a more uniform approach to planning and reporting simulation studies using the ADEMP framework [41], and hopefully due to this, summarising results of multiple simulation studies will be easier in the future, once the implementation of these guidelines has filtered through to systematic review level. In this paper, earlier guidance was used to help guide the inclusion criteria and quality assessment of papers as they are less recent and less specific than the ADEMP framework [14]. However, this still has the potential to induce bias, since some papers included in this review were written prior to this guidance in 2006.

Finally, it is important to note that many of the methods examined in this review could also be applied to deal with confounding in observational studies, which is analogous to the issue of non-compliance in RCTs. However, the focus of this review is the latter issue and hence, statistical methods to handle any potential confounding that is not related to non-compliance were not considered. Extending the scope of the review to cover this wider subject would have been infeasible. For this same reason, non-inferiority and equivalence trials were excluded, but it would be certainly be useful to also assess the methods that have been investigated in these settings, as they may differ to the ones primarily considered in this paper.

## Conclusions

Participant’s compliance with their randomised intervention in RCTs is rarely perfect and may impact the validity of trial results. There is a large body of research focussing on making causal inferences in RCTs when participants do not comply with the original protocol. However, much of the focus is on the development of novel methods and extensions or improvement of existing methods. Fewer papers direct their attention to comparison of these methods in a range of scenarios in order to fully evaluate them, and hence, there is little evidence available to applied researchers working in clinical trials in order to inform their decisions in a practical setting. The objective of this systematic review was to identify methods papers which aim to compare the performance of various existing methods to estimate a treatment effect in the presence of non-compliance in simulation studies.

This review identified a lack of comparison of specialised G-methods that allow for time-varying non-compliance, although these methods appear to be compared more thoroughly in literature related to treatment switching. Whilst this is also an important methodological issue, is may refer to a separate setting and it is not clear whether the results of these papers are generalisable to the definition of non-compliance considered within this paper. With the current state of the literature, it is difficult to make specific recommendations about which methods are most appropriate to use to deal with non-compliance, given the differences between the studies included in this review. More simulation studies are needed that compare a range of relevant methods, in order for replication of results and a consensus in recommendations to be achieved.

### Electronic supplementary material

Below is the link to the electronic supplementary material.

Supplementary Material 1

Supplementary Material 2

Supplementary Material 3

Supplementary Material 4

## Data Availability

Not applicable.

## Abbreviations

AT: As treated
CACE: Complier average causal effect
CALM: Causal accelerated life model
CHARM: Causal hazard ratio adjustment regression model
CI: Confidence interval
CL: Cluster level
CP: Coverage probability
C-Prophet: Compliers proportional hazards effect of treatment
ER: Exclusion restriction
GEE: General estimating equations
HLM: Hierarchical linear model
HR: Hazard ratio
HTA: Health technology assessment
ICC: Intra-cluster correlation coefficient
IPCW/IPTW: Inverse probability of censoring/treatment weighting
IP-weighted: Inverse probability weighted
ITT: Intention to treat
IV: Instrumental variable
LATE: Local average treatment effect
LTGM: Latent treat grizzle model
MC: Monte Carlo
MLE: Maximum likelihood estimation
MSM: Marginal structural model
NPCB: Non-parametric causal bound
OLS: Ordinary least squares
OR: Odds ratio
PP: Per protocol
RCT: Randomised controlled trial
(R)MSE: (Root) mean squared error
RPSFTM: Rank preserving structural failure time model
SD: Standard deviation
SE: Standard error
SSDF: Small sample degrees of freedom
2SLS/TSLS: Two-stage least squares
2SPS: Two-stage predictor substitution
2SRI: Two-stage residual inclusion
