# Supplementary material for: A systematic review of simulation studies which compare existing statistical methods to account for non-compliance in randomised controlled trials
Source: BMC Med Res Methodol. 2023 Dec 16;23:300. doi: 10.1186/s12874-023-02126-w (PMC10724933; doi:10.1186/s12874-023-02126-w)
Supplement: Supplementary file 2 — Supplementary Material 2 [file 12874_2023_2126_MOESM2_ESM.pdf]

List of papers identified during full-text review which were deemed to fit the inclusion criteria but specifically in relation to treatment switching rather than non-compliance to the protocol.

Latimer NR, Abrams KR, Lambert PC, Crowther MJ, Wailoo AJ, Morden JP, Akehurst RL, Campbell MJ. Adjusting for treatment switching in randomised controlled trials - A simulation study and a simplified two-stage method. Stat Methods Med Res. 2017 Apr;26(2):724-751. doi: 10.1177/0962280214557578. Epub 2014 Nov 21. PMID: 25416688.

Latimer NR, Abrams KR, Lambert PC, Morden JP, Crowther MJ. Assessing methods for dealing with treatment switching in clinical trials: A follow-up simulation study. Stat Methods Med Res. 2018 Mar;27(3):765-784. doi: 10.1177/0962280216642264. Epub 2016 Apr 25. PMID: 27114326.

Latimer NR, Abrams KR, Siebert U. Two-stage estimation to adjust for treatment switching in randomised trials: a simulation study investigating the use of inverse probability weighting instead of re-censoring. BMC Med Res Methodol. 2019 Mar 29;19(1):69. doi: 10.1186/s12874-019-0709-9. PMID: 30935369; PMCID: PMC6444622.

Morden JP, Lambert PC, Latimer N, Abrams KR, Wailoo AJ. Assessing methods for dealing with treatment switching in randomised controlled trials: a simulation study. BMC Med Res Methodol. 2011 Jan 11;11:4. doi: 10.1186/1471-2288-11-4. PMID: 21223539; PMCID: PMC3024998.

Ouwens M, Hauch O, Franzén S. A Validation Study of the Rank-Preserving Structural Failure Time Model: Confidence Intervals and Unique, Multiple, and Erroneous Solutions. Med Decis Making. 2018 May;38(4):509-519. doi: 10.1177/0272989X18765175. Epub 2018 Apr 1. PMID: 29607730.

Malihe Safari, Habib Esmaeili, Hossein Mahjub & Ghodratollah Roshanaei (2021) Estimation of treatment effect in presence of noncompliance with early or late switching: a simulation study, Communications in Statistics - Simulation and Computation, DOI: 10.1080/03610918.2021.1970183

Xu J, Liu G, Wang B. Bias and Type I error Control in Correcting Treatment Effect for Treatment Switching Using Marginal Structural Models in Phase III Oncology Trials. J Biopharm Stat. 2022 Nov 2;32(6):897-914. doi: 10.1080/10543406.2022.2058524. Epub 2022 Jun 3. PMID: 35656809.
