# Supplementary material for: A systematic review of simulation studies which compare existing statistical methods to account for non-compliance in randomised controlled trials
Source: BMC Med Res Methodol. 2023 Dec 16;23:300. doi: 10.1186/s12874-023-02126-w (PMC10724933; doi:10.1186/s12874-023-02126-w)
Supplement: Supplementary file 3 — Supplementary Material 3 [file 12874_2023_2126_MOESM3_ESM.pdf]

Search number	Query	Filters	Search Details	Results
18	#6 AND #17	English, MEDLINE	((("computer simulation/methods"[MeSH Terms] AND ("medline"[Filter] AND "english"[Language])) OR ("computer simulation/statistics and numerical data"[MeSH Terms] AND ("medline"[Filter] AND "english"[Language])) OR ("simulat*"[Text Word] AND ("medline"[Filter] AND "english"[Language])) OR ("simulation study"[Text Word] AND ("medline"[Filter] AND "english"[Language])) OR ("Monte Carlo simulation"[Text Word] AND ("medline"[Filter] AND "english"[Language]))) AND ("medline"[Filter] AND "english"[Language]) AND ((("Medication Adherence"[MeSH Terms] AND ("medline"[Filter] AND "english"[Language])) OR ("Patient Compliance"[MeSH Terms] AND ("medline"[Filter] AND "english"[Language])) OR ("Treatment Switching"[MeSH Terms] AND ("medline"[Filter] AND "english"[Language])) OR ("non-compliance"[Text Word] AND ("medline"[Filter] AND "english"[Language])) OR ("noncompliance"[Text Word] AND ("medline"[Filter] AND "english"[Language])) OR ("non-adherence"[Text Word] AND ("medline"[Filter] AND "english"[Language])) OR ("nonadherence"[Text Word] AND ("medline"[Filter] AND "english"[Language])) OR ("non-compliance"[Text Word] AND ("medline"[Filter] AND "english"[Language])) OR ("non-adherence"[Text Word] AND ("medline"[Filter] AND "english"[Language])) OR ("Treatment Switching"[Text Word] AND ("medline"[Filter] AND "english"[Language]))) AND ("medline"[Filter] AND "english"[Language]))) AND ((medline[Filter]) AND (english[Filter]))	845
17	#7 OR #8 OR #9 OR #10 OR #11 OR #12 OR #13 OR #14 OR #15 OR #16	English, MEDLINE	(("Medication Adherence"[MeSH Terms] AND ("medline"[Filter] AND "english"[Language])) OR ("Patient Compliance"[MeSH Terms] AND ("medline"[Filter] AND "english"[Language])) OR ("Treatment Switching"[MeSH Terms] AND ("medline"[Filter] AND "english"[Language])) OR ("non-compliance"[Text Word] AND ("medline"[Filter] AND "english"[Language])) OR ("noncompliance"[Text Word] AND ("medline"[Filter] AND "english"[Language])) OR ("non-adherence"[Text Word] AND ("medline"[Filter] AND "english"[Language])) OR ("nonadherence"[Text Word] AND ("medline"[Filter] AND "english"[Language])) OR ("non-compliance"[Text Word] AND ("medline"[Filter] AND "english"[Language])) OR ("non-adherence"[Text Word] AND ("medline"[Filter] AND "english"[Language])) OR ("Treatment Switching"[Text Word] AND ("medline"[Filter] AND "english"[Language]))) AND ((medline[Filter]) AND (english[Filter]))	90,094
16	"treatment switching"[tw]	English, MEDLINE	("treatment switching"[Text Word]) AND ((medline[Filter]) AND (english[Filter]))	250
15	"non adherence"[tw]	English, MEDLINE	("non adherence"[Text Word]) AND ((medline[Filter]) AND (english[Filter]))	5,648

14	"non compliance"[tw]	English, MEDLINE	("non compliance"[Text Word]) AND ((medline[Filter]) AND (english[Filter]))	4,344
13	"nonadherence"[tw]	English, MEDLINE	("nonadherence"[Text Word]) AND ((medline[Filter]) AND (english[Filter]))	11,070
12	"non-adherence"[tw]	English, MEDLINE	("non-adherence"[Text Word]) AND ((medline[Filter]) AND (english[Filter]))	5,648
11	"noncompliance"[tw]	English, MEDLINE	("noncompliance"[Text Word]) AND ((medline[Filter]) AND (english[Filter]))	10,010
10	"non-compliance"[tw]	English, MEDLINE	("non-compliance"[Text Word]) AND ((medline[Filter]) AND (english[Filter]))	4,344
9	"Treatment Switching"[Mesh]	English, MEDLINE	("Treatment Switching"[MeSH Terms]) AND ((medline[Filter]) AND (english[Filter]))	19
8	"Patient Compliance"[Mesh]	English, MEDLINE	("Patient Compliance"[MeSH Terms]) AND ((medline[Filter]) AND (english[Filter]))	78,571
7	"Medication Adherence"[Mesh]	English, MEDLINE	("Medication Adherence"[MeSH Terms]) AND ((medline[Filter]) AND (english[Filter]))	23,935
6	#1 OR #2 OR #3 OR #4 OR #5	English, MEDLINE	(("computer simulation/methods"[MeSH Terms] AND ("medline"[Filter] AND "english"[Language])) OR ("computer simulation/statistics and numerical data"[MeSH Terms] AND ("medline"[Filter] AND "english"[Language])) OR ("simulat*"[Text Word] AND ("medline"[Filter] AND "english"[Language])) OR ("simulation study"[Text Word] AND ("medline"[Filter] AND "english"[Language])) OR ("Monte Carlo simulation"[Text Word] AND ("medline"[Filter] AND "english"[Language]))) AND ((medline[Filter]) AND (english[Filter]))	579,047
5	"Monte Carlo simulation"[tw]	English, MEDLINE	("Monte Carlo simulation"[Text Word]) AND ((medline[Filter]) AND (english[Filter]))	8,943
4	"simulation study"[tw]	English, MEDLINE	("simulation study"[Text Word]) AND ((medline[Filter]) AND (english[Filter]))	12,567
3	simulat*[tw]	English, MEDLINE	("simulat*"[Text Word]) AND ((medline[Filter]) AND (english[Filter]))	579,037
2	"Computer Simulation/statistics and numerical data"[Mesh]	English, MEDLINE	("computer simulation/statistics and numerical data"[MeSH Terms]) AND ((medline[Filter]) AND (english[Filter]))	867
1	"Computer Simulation/methods"[Mesh]	English, MEDLINE	("computer simulation/methods"[MeSH Terms]) AND ((medline[Filter]) AND (english[Filter]))	1,322
