# Supplementary material for: A systematic review of simulation studies which compare existing statistical methods to account for non-compliance in randomised controlled trials
Source: BMC Med Res Methodol. 2023 Dec 16;23:300. doi: 10.1186/s12874-023-02126-w (PMC10724933; doi:10.1186/s12874-023-02126-w)
Supplement: Supplementary file 4 — Supplementary Material 4 [file 12874_2023_2126_MOESM4_ESM.docx]

**Assessing the performance of statistical methods to account for non-compliance in randomised controlled trials: a systematic review protocol**

INTRODUCTION

Non-compliance occurs when participants in clinical trials do not adhere to the intervention group that they were originally randomised to. This may refer to individuals dropping out or missing certain elements of the treatment or intervention, but can also include control group participants diverting to the experimental or some other treatment. Although various terms have been used to describe this issue throughout current literature, this protocol will stick to the phrase ‘non-compliance’ in order to encompass these different scenarios and represent any departure from randomised treatment.

Non-compliance has the potential to reduce the power of an intention-to-treat (ITT) analysis [1], in which participants are analysed based on their allocated group, irrespective of the treatment they actually receive. It also means that, despite randomisation, it cannot be guaranteed that the relationship between treatment and outcome is unconfounded [2]. These limitations are summarised nicely by Sagarin et al (2014), who remark that “non-compliance is difficult to model and perilous to ignore” [3].

Despite this clear issue, there is a distinct lack of guidance surrounding the reporting and handling of non-compliance within RCTs, with the 2010 CONSORT guidelines stating that “the simple way to deal with any protocol deviations is to ignore them” [4]. These guidelines recommend reporting per protocol (PP) methods, where non-compliers are excluded from analysis, in addition to reporting of the ITT effect. Whilst this approach does attempt to account for compliance behaviours, it relies on the assumption that the now non-randomised groups are comparable which is unlikely to hold and may result in estimates of the treatment effect that are subject to selection bias [3].

Previous reviews have identified statistical methods to deal with this issue in non-inferiority trials [5] and in a time-to-event and HTA context [6]. Additionally, Mostazir et al conducted a methodological review of RCTs in order to assess which methods are most commonly used to handle non-adherence to the protocol [7]. Methods identified across these reviews included principal stratification methods such as instrumental variables and g-estimation methods such as marginal structural models with inverse probability of censoring weighting and rank-preserving structural failure time models. Whilst these reviews provide a useful summary of the existing methods to deal with the issue of non-compliance in a range of contexts, they provide little information about the performance of these methods. Indeed, all three papers concluded that further work is required to assess and compare the performance of these methods [5-7].

A general search found there to be wide and varied literature on methods to account for non-compliance in RCTs. Many of these papers proposed new methods in order to address a specific scenario and evaluated their finite sample performance under simulation. Whilst using simulation in this manner is common practice, Boulesteix et al (2013) argue that these papers should be treated with caution, since these simulations may be prone to “inventor bias” [8]. Pawel et al (2022) also recently demonstrated how it is relatively easy to prove new methods to be optimal using simulation studies [9].

Applying the idea of the ‘phases of statistical methodology research’ framework recently proposed by Heinze et al (2023), it appears that many of these papers could be described as covering a ‘phase I/II’ level of research. Heinze et al noted that many methods are proposed without ever being fully investigated and introduced their framework in order to put more weight on studies that conduct carefully planned method comparisons of explore the empirical properties of methods in a wide range of scenarios [10]. The aim of this systematic review is to identify such papers in the context of statistical methodologies to deal with non-compliance to the intervention protocol. Collating the results from authors that have used simulations to assess non-compliance adjusting methods in this manner will allow for the methods most widely considered to be compared and evaluated based on evidence from a number of studies and overall inferences made about this area of research. A natural drawback of simulation studies is that, whilst they allow for precise simulation conditions relevant to the problem of interest to be specified, this results in poor external validity. This issue is improved by collating the results of multiple simulation studies which are likely to consider a range of scenarios, which in turn also combats any potential for misinterpretation of individual studies [11]. Not only will this approach produce a summary of the performance of some key methods, it will also provide a better picture of the landscape and progress of research in this area.

OBJECTIVES

This systematic review aims to identify all methodological papers that have evaluated and compared a number of methods to deal with non-compliance in RCTs using a detailed simulation study. The results of this review could be used in order to identify gaps in current research, inform further work or provide guidance for applied researchers wanting to consider compliance within their analysis. The goal of this review is to address the following questions:

1. Which methods to deal with non-compliance have been most thoroughly investigated by researchers undertaking simulation studies in this area and how do these methods perform under various scenarios?
2. What does this tell us the research deficits in this area? (E.g. which methods need to be evaluated more rigorously?)

METHODS

This protocol follows the Preferred Reporting Items for Systematic Reviews and Meta-Analyses (PRISMA) Protocol checklist for systematic review protocols [12]. The review itself will also be reported using the most recent version of the PRISMA guidelines [13].

*Search strategy and information sources*

The search strategy will be developed by the primary author, with the assistance of a librarian specialising in medical and health information sources, and peer reviewed by other authors prior to commencement of the review. The online databases MEDLINE, Scopus, Web of Science and MathSciNet will be searched from conception to 30^th^ November 2022 using an appropriate combination of keywords based on the topic of interest. The main search terms used, which are included in Appendix A, was written for MEDLINE and will be adapted for the other databases. Full search strategies will be included in the Appendix of the full review paper. Searches will be limited to publications that are readily available in the English language and all search results will be sorted and exported using EndNote 20.3. Selected studies will have their reference list checked for further studies that may fit the eligibility criteria. It is not relevant to search for ongoing studies in this review.

*Eligibility criteria*

Papers to be included within this review should adhere to the following criteria:

| **Inclusion criteria** | **Exclusion criteria** |
| --- | --- |
| Peer-reviewed methodological papers whose focus is to compare two or more existing methods under a sufficient simulation study* | Non-peer reviewed articles, books or book chapters, theses or other grey literature such as conference proceedings; |
| The methodological topic of interest is non-compliance to the randomised intervention, which may be by participants in the intervention or control groups. This compliance could be described as all-or-nothing or time varying/partial; | Papers that focus on issues such as drop-out and missing data or the combination of these issues with non-compliance; |
| The methods considered are explicitly applied to account for non-compliance in the setting of a superiority RCT; | Papers whose focus is a novel method rather than comparison of existing methods (e.g. that describe/reference a current method and propose an extension to it or propose a new method) |
| Papers published from databases inception to date; and | Papers that focus on an observational setting; |
| Papers published in the English language. | Papers that consider a non-inferiority or equivalence setting |
|  | Methods based on aggregated data such as meta-analysis; or |
|  | Theoretical papers with no application or assessment of the method via simulation. |

*Note that it is difficult to be fully objective here, but this criterion will be based on the following:

- The simulation study clearly states its objectives and gives a description of how the simulation was conducted/the nature of the simulated data
- The simulation study compares at least two existing methods that aim to account for non-compliance and estimate a point estimate of the intervention effect.
- Existing methods refers to those that have not been proposed in the paper of interest and the authors have referenced previous work when describing the method.
- The authors consider several non-compliance scenarios, such as varying the proportion or type of non-compliance.
- Amongst performance measures, at least the bias of methods is reported or can be easily deduced.

These criteria were based on guidance for reporting simulation studies by Burton et al (2006) and a scoping review of targeted papers [14] .

*Screening*

Titles, abstracts and authors from all search results will be exported into Covidence^[[1]](#footnote-1)^, as well as the date and location of publication, with any duplicate papers deleted [15]. Initially, titles and abstracts will be screened to assess whether they meet the inclusion criteria specified. Full text screening will then be carried out. If decisions cannot be made at this stage, then the authors of any remaining papers will be contacted in order to settle this issue. All articles found during the search will be screened by both the primary author and a second independent reviewer. Any disagreement will be resolved either by discussion or using a third independent reviewer. Excluded studies at the full text stage will be presented in a table, which will be included in the review paper, in order to detail their characteristics and reason for exclusion.

*Data extraction*

Extraction of data from selected studies will be performed by the primary author using a predefined extraction form, with input from a secondary independent reviewer in the same manner as during article screening. The data to be extracted is based upon a checklist of elements to consider during the design of simulation studies, recommended by Burton et al (2006) [14]. Again, any differences will be discussed and resolved using a third independent reviewer if necessary. A pilot data extraction will be carried out prior to the main analysis in order to test the form created.

Data to be extracted includes:

- General information: Title, Lead author with correspondence, Year, Country, Journal
- Motivation and aims of the study
- Specific type of non-compliance considered (e.g., just by treatment group, treatment switching, all-or-nothing, etc.)
- Estimand of interest
- Methods considered
- Method(s) used to simulate data, including any values assumed (e.g. true effect size)
- Method(s) used for generation of compliance status
- Scenarios/simulation conditions considered
- Number of simulations performed
- Results of simulation study, specifically the performance measures reported (such as bias, coverage, etc.) or the trends in these values across simulation conditions if not explicitly reported
- Main conclusions/recommendations of the authors based on these results

*Outcomes*

The key performance measures reported from the simulation study, such as bias, coverage and empirical and model based standard errors will be used in part to compare and draw conclusions about the methods considered. If these are not directly reported, inferences will be based on how they vary across performance conditions. Authors will be contacted if sufficient information about such performance methods is not detailed in the paper. Due to the range of measures that may be reported across studies, an attempt will be made to transform results onto the same scale [11]. Where this is not possible, it will be ensured that direct comparisons are predominantly made and the differences in performance measures are taken into account.

No further specific quantitative outcomes will be targeted, due to the aims of the review in producing a more narrative summary of methods to deal with non-compliance and the conclusions that have been made by authors of simulation studies themselves on the matter.

Considerations of this nature will include:

- Types of settings considered (if possible to group based on non-compliance type, outcome type, etc.)
- Method(s) most commonly considered within these simulation studies
- General inferences/recommendations related to these methods
- Scenarios considered and any potentially overlooked

*Risk of bias in individual studies*

The quality of the studies included within this review is an important consideration as this will impact the reliability of any conclusion made. No defined tool exists to assess the risk of bias in simulation studies. The following questions are based on areas where it would be possible for bias to occur and will be examined within each study.

- Do the authors justify the sample size used for the number of simulations?
- Do the authors justify the values selected within the generation of data or any other variables considered?
- Are any assumptions made throughout the study, such as in the data generation model, and are these justified?
- To what extent are the conclusions made by the authors supported by the results of the simulation study?

*Patient and public involvement*

Due to the key objectives of this systematic review being to inform other methodological and applied statistical researchers, it was not appropriate or possible to involve patients or the public in the planning, design, conduct, reporting or dissemination of this protocol or main review paper.

*Data synthesis*

A flow chart of studies found during the search which details how many were eventually included in the review will be produced. For papers that meet the inclusion criteria, the relevant data will be collected using the data extraction form and reported in an appropriate table that allows for a summary of the most important aspects of each study to be presented together. From this table, overall inferences will be made based on the cumulative evidence found. Risk of bias information will also be reported in a suitable table and used to assess the strength of inferences made based on each study. It would not be possible to assess publication bias across studies using a direct measure.

DISCUSSION

Non-compliance to a treatment or intervention is an issue that often arises in RCTs and may impact the power of analyses such as ITT. Previous research has found there to be an abundance of literature proposing and discussing methods to account for non-compliance within analysis. However, these methods need to be scrutinised and tested in a range of realistic settings before applied researchers may feel confident that there is sufficient evidence to use them routinely in practice.

The aim of this systematic review is to provide an overview of the methods to deal with non-adherence to intervention protocol that have been most scrutinised by methodological researchers by assessing and comparing them under detailed simulation studies. In addition, it is anticipated that the review will address any other issues related to simulation studies in this area, such as the appropriate generation of compliance status within the data in order to mimic real world scenarios.

*Strengths and limitations of this study*

- The reporting of this study will adhere to the PRISMA guidelines where appropriate.
- The narrative nature of this review ensures that it is possible to address a number of questions related to the issue of non-compliance and simulation studies carried out within this area simultaneously.
- It is expected that this review of simulation studies will provide evidence about which methods are the most appropriate to deal with non-compliance in a range of contexts, based on which methods have been sufficiently assessed. This may encourage increased uptake of such methods as well as highlighting the need for further methodological research in this area.
- It cannot be ensured that all existing methods to account for non-compliance within analysis will be covered within this review, as they may not have been considered within a relevant simulation study. In fact, it is very unlikely that all methods will be included, but this is still a useful result, as it shows which methods need to be examined in greater detail/further work.
- The reporting of simulation studies may vary considerably across the papers included in this review, making the synthesis of results more complex and descriptive.

REFERENCES

1. White, I.R., *Uses and limitations of randomization-based efficacy estimators.* Statistical Methods in Medical Research, 2005. **14**(4): p. 327-347.

2. Agbla, S.C. and K. DiazOrdaz, *Reporting non-adherence in cluster randomised trials: A systematic review.* Clin Trials, 2018. **15**(3): p. 294-304.

3. Sagarin, B.J., et al., *Treatment noncompliance in randomized experiments: statistical approaches and design issues.* Psychol Methods, 2014. **19**(3): p. 317-33.

4. Moher, D., et al., *CONSORT 2010 explanation and elaboration: updated guidelines for reporting parallel group randomised trials.* BMJ, 2010. **340**: p. c869.

5. Dodd, M., et al., *Statistical methods for non-adherence in non-inferiority trials: useful and used? A systematic review.* BMJ Open, 2022. **12**(1): p. e052656.

6. Alshreef, A., et al., *Statistical Methods for Adjusting Estimates of Treatment Effectiveness for Patient Nonadherence in the Context of Time-to-Event Outcomes and Health Technology Assessment: A Systematic Review of Methodological Papers.* Med Decis Making, 2019. **39**(8): p. 910-925.

7. Mostazir, M., et al., *An overview of statistical methods for handling nonadherence to intervention protocol in randomized control trials: a methodological review.* J Clin Epidemiol, 2019. **108**: p. 121-131.

8. Boulesteix, A.L., S. Lauer, and M.J. Eugster, *A plea for neutral comparison studies in computational sciences.* PLoS One, 2013. **8**(4): p. e61562.

9. Pawel, S., L. Kook, and K. Reeve, *Pitfalls and Potentials in Simulation Studies*. 2022.

10. Heinze, G., et al., *Phases of methodological research in biostatistics-Building the evidence base for new methods.* Biom J, 2023: p. e2200222.

11. LeBeau, B., *Research synthesis and meta‐analysis of Monte Carlo studies: the best of two worlds.* Campbell Systematic Reviews, 2017. **13**(1): p. 1-9.

12. Shamseer, L., et al., *Preferred reporting items for systematic review and meta-analysis protocols (PRISMA-P) 2015: elaboration and explanation.* BMJ, 2015. **350**: p. g7647.

13. Page, M.J., et al., *The PRISMA 2020 statement: an updated guideline for reporting systematic reviews.* BMJ, 2021. **372**: p. n71.

14. Burton, A., et al., *The design of simulation studies in medical statistics.* Stat Med, 2006. **25**(24): p. 4279-92.

15. *Covidence systematic review software*. Veritas Health Innovation.

1. Covidence is a web-based collaboration software platform that streamlines the production of systematic and other literature reviews. Covidence uses iterative product development processes, and therefore we do not use version numbers or years. [↑](#footnote-ref-1)
